# Efficacy of Inelastic Compression Devices with a High Stiffness in Older Breast Cancer Survivors with Moderate and Severe Lymphedema: A Prospective Interventional Study

**DOI:** 10.3390/jcm15187310

**Published:** 2026-09-20

**Authors:** Katarzyna Ochałek, Ewelina Matusik, Zbigniew Szygula

**Affiliations:** 1 Faculty of Motor Rehabilitation, Institute of Clinical Rehabilitation, University of Physical Culture, 31-571 Kraków, Poland; 2 Lymphedema Clinic, St. Lazarus Hospice, 31-380 Kraków, Poland; 3 Department of Sports Medicine and Human Nutrition, Faculty of Physical Education and Sport, University of Physical Culture, 31-571 Kraków, Poland

**Keywords:** breast cancer, lymphedema, compression therapy, multi-layer bandaging, adjustable compression wraps

## Abstract

**Objectives:** The study aimed to assess the effectiveness, physical functioning and comfort of decongestive adjustable compression wraps (ACW, Circaid^®^ reduction kit), the essential ACW (Circaid^®^ juxtafit™ essentials) and MLB (Multi-layer bandaging) among patients with breast cancer-related lymphedema (BCRL), divided into a moderate (MG, *n* = 28) and severe group (SG, *n* = 27), based on BCRL severity. **Methods:** The treatment lasted 10 days and was carried out over a two-week period. In the first week, all patients were treated by experienced physiotherapists. In the second week, the protocol was continued at home. Measurements, including limb volumes, physical functioning and disease-related symptoms, were performed prior to the intervention (at baseline), as well as following one and two weeks of treatment. Compression comfort was assessed in the groups after one and two weeks. **Results:** In both groups, a significant reduction in affected limb volume was found after one week (*p* < 0.001). The pressure under the decongestive ACW device was significantly lower (*p* < 0.001) in comparison to the essential ACW and MLB. The affected limb joint mobility in the decongestive ACW group was significantly higher after the second week in the SG (*p* = 0.023). Although minor complications of compression were observed less frequently with MLB than with ACWs, compression comfort was comparable between the groups. **Conclusions:** Both ACWs may represent feasible alternatives to multi-layer bandaging during the intensive phase of complex decongestive therapy in selected patients.

## 1. Introduction

Breast cancer-related lymphedema (BCRL) is a progressive condition that affects upper limb function and can cause detrimental effects on the quality of life of an individual [1]. There are several stages of clinical development according to the International Society of Lymphology (ISL), ranging from subclinical stasis (stage 0) to severe lymphedema (LE) with a tendency towards complications and limb deformity (stage 3) [2]. Within each stage, functional severity assessment uses percentage of excess volume (PEV) to categorise severity as minimal (5–20% PEV), moderate (20–40%), or severe (>40% increase) [2].

Pathological changes in the arm during the development of LE reduce lymph flow and alter the morphology of lymphatic vessels, even during the latent or subclinical stages, when obvious oedema is absent. Over time, collecting lymphatic vessels exhibit increased inflammation and abnormal smooth muscle cell coverage, leading to a thickened vessel wall. Dilation also occurs, resulting in arm LE and fibrosis [3]. Moderate to severe stages of BCRL consist of skin structure changes. The continuous expansion of fibrotic tissue and fat deposition affects the thickness of the skin and subcutaneous tissue, leading to hardening of the skin [1].

Identifying the most effective treatment options for every stage of lymphedema is crucial. Treatment should not only be aimed at reducing the extent of lymphedema and disease-related symptoms, but also at preventing complications that decrease physical functioning [4]. Compression therapy (CT), widely recommended by the ISL using both inelastic multi-layer bandaging (MLB) and compression garments (CG), still remains the most efficient component of complex decongestive therapy (CDT) at every stage of LE clinical advancement [2].

For patients with the initial signs of BCRL and mild LE, general measures such as compression garment use (a component of compression therapy) and functional exercise in compression, maintaining proper body weight, and skin care are the first line of treatment [5]. Early treatment, before the development of fat accumulation and fibrosis, should be a primary goal in the treatment of BCRL. Research indicates that the use of compression at the initial stage of the disease may prevent progression of lymphedema and its complications [5].

Therapeutic management, especially for the elderly in advanced and chronic cases of LE, is typically more difficult and challenging. Rehabilitation strategies should not only be aimed at reducing the extent of LE, but also at improving or preserving arm functionality to enhance quality of life (QoL) in BCRL survivors [6]. The relationship between BCRL severity and functional decline underscores the need for carefully prescribed physical exercise with appropriate compression devices to prevent symptom exacerbation [7]. MLB has been shown as highly effective in reducing limb volume at the initial phase of CDT [8,9,10,11]. However, this method may be less suitable for long-term therapeutic use in older and severe LE populations due to its complexity and limited practicality in routine self-management. Adjustable compression wraps (ACWs) have been proposed as an alternative to the commonly used MLB and have proven to be effective and well-tolerated mainly in vascular disorders of the lower limbs [12,13,14,15,16,17]. These results cannot be directly extrapolated to other locations, especially the upper limb in BCRL.

Only a limited number of studies have been carried out on the effect of ACWs in BCRL survivors, and different types of ACW devices with MLBs were compared in them [18,19,20]. There is a lack of research establishing which type of inelastic ACW device is the most effective and functional in older patients and those with more advanced BCRL.

The objective of the present study was to assess the effectiveness in limb volume reduction, physical functioning and comfort of three inelastic compression types: decongestive ACW (Circaid^®^ reduction kit), the essential ACW (Circaid^®^ juxtafit™ essentials) and MLB in the moderate and severe stages of BCRL according to the ISL during the intensive phase of therapy.

## 2. Materials and Methods

In the period between January 2025 and March 2026, 55 women following breast cancer treatment, who fulfilled the inclusion criteria (LE, ≥20% PEV, positive pitting skin sign, with no signs of active cancer, venous thrombosis or previous compression), were admitted to the Lymphedema Clinic. Based on lymphedema severity, they were non-randomly assigned to the moderate group (MG, stage II, 20–40% PEV, *n* = 28) or the severe group (SG, stage III, ≥40% PEV, *n* = 27). The first patient was enrolled in July 2025 (i.e., after registration). Within each of the two severity strata, patients were allocated to one of three parallel treatment subgroups based on chronological sequence: (1) decongestive ACW (decongestive ACW-MG, *n* = 10 vs. decongestive ACW-SG, *n* = 9), (2) essential ACW (essential ACW-MG, *n* = 9 vs. essential ACW-SG, *n* = 9), (3) MLB (MLB-MG, *n* = 9 vs. MLB–SG, *n* = 9). Once assigned, the treatment was delivered concurrently across six total subgroups (3 compression modalities × 2 severity groups).

**Figure 1:**
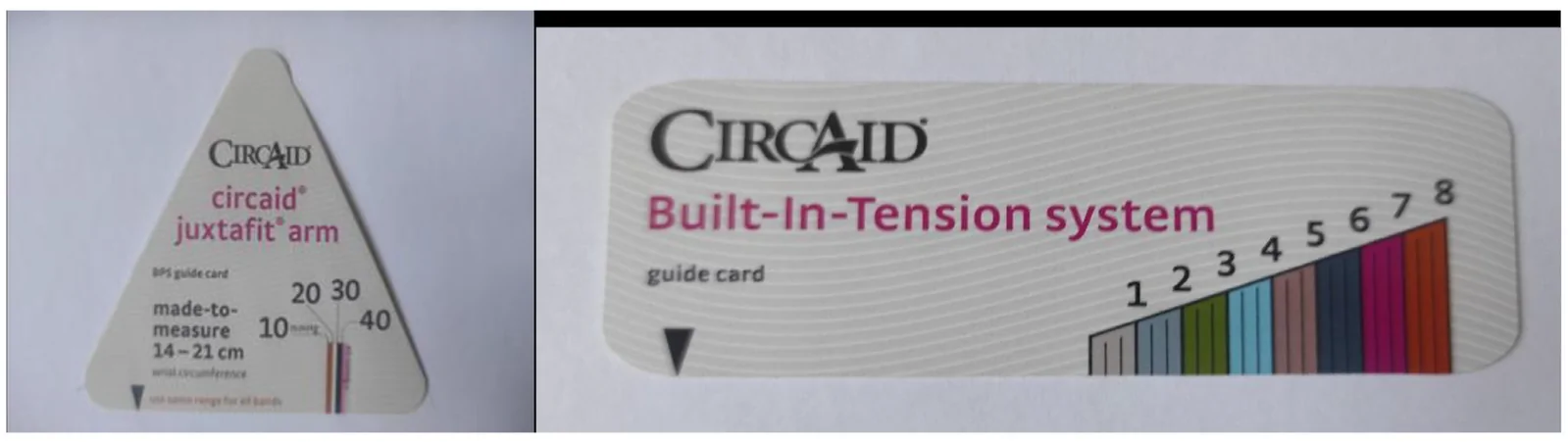
Colour scale cards for self-monitoring the applied compression force under essential ACW and decongestive ACW devices.

Both types—decongestive ACWs (Circaid^®^ reduction kit, Medi-Bayreuth, Germany) and essential ACWs (Circaid^®^ juxtafit™ essentials, Medi-Bayreuth, Germany)—were fitted based on individual limb measurements. Both systems are interlacing (a technique in which layers of the wrap are applied in opposing directions, creating a criss-cross pattern). They are characterised by an inelastic material with high wall stability for high working pressure and demonstrate readjustability of the individual bands. This limits pressure loss and thus sliding down of the garment. The main difference between the two types of wraps is that decongestive ACWs (circaid reduction) offer an additional option of trimming the material which reduces the size of the garment as the limb volume decreases. Both ACW devices have a controlled system for putting on and checking the applied compression force (colour scale cards, Figure 1) that allows patients to achieve the appropriate pressure above 23 mmHg. The third type of compression, MLB, comprises four 8, 10 and 12 cm wide layers, and is made of a short-stretch material (Rosidal K, Lohmann and Rauscher, Germany), with cotton tubes and foam padding underneath.

**Figure 2:**
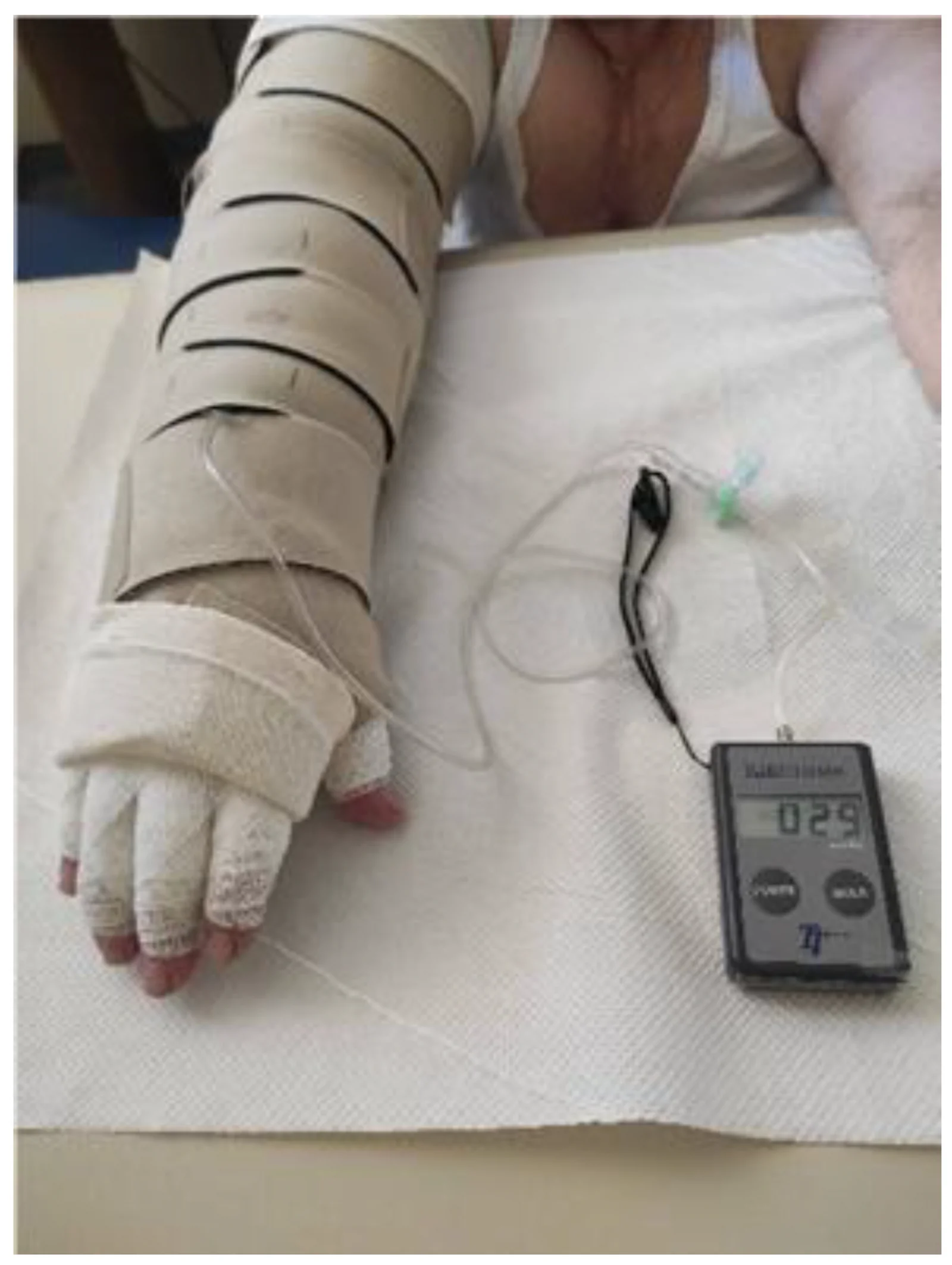
Measurement of interface pressure under ACW device.

The treatment schedule for all groups lasted 10 days (two weeks, from Monday to Friday). In the first week, the patients were treated every day by two experienced physiotherapists. Prior to the consecutive therapeutic session, the compression products were removed for several minutes in order to refresh and moisturise the skin. Apart from the various types of compression, all patients underwent a unified exercise programme, including aerobic exercises of the upper limbs combined with deep breathing for 15 min during each session. The compression material in the decongestive ACWs was individually trimmed at the end of the first week of therapy. During the first week, all patients and their caregivers were also provided with comprehensive education on applying the compression devices and exercise in compression. The physiotherapist first demonstrated to patients how to apply ACWs or MLBs, and then observed and supervised each of them during application. In the second week, the use of ACWs and MLBs was continued by the patients themselves at home. Caregivers were actively involved in assisting with the application of the compression devices or bandaging. Patients were asked to maintain the adjustable compression wraps or multi-layer bandaging for 24 h, even during the weekend (Saturday and Sunday), and to continue the learned exercise programme. Interface pressure was measured under compression via the Kikuhime device (Kikgel, Ujazd, Poland) on the dorsal part of the forearm on the first day of therapy to ensure a comparable pressure value in the compression systems (Figure 2). To ensure safety and good treatment tolerability, the applied compression force (via the Kikushime device or a colour card) was checked daily by a physiotherapist for all patients during the first week. Monitoring the applied compression force (via colour scale cards) by the patients and their caregivers to obtain similar interface pressure under the ACW device was recommended during the second week of self-management. For self-multilayer bandaging, patients were advised to use the same number of bandages and apply similar tension to achieve identical sub-bandage pressure as when bandaged by a physiotherapist in the clinic.

Measurements in all subjects, including limb volumes, physical functioning and disease-related symptoms, were performed prior to the intervention, as well as after one and two weeks of treatment. Calculation of limb volumes (based on circumferential measurements every 4 cm) was carried out using a simplified frustum (summed truncated cone) formula.

To quantify lymphedema, which functions independently of the opposite arm and accounts for fluctuations in patient weight, we additionally used the WAC formula (weight-adjusted volume change calculated as: WAC = (*A*_2_*BMI*_1_/*BMI*_2_*A*_1_) − 1, where *A*_1_ and *A*_2_ are arm volumes of the treated arm at baseline and after two weeks, and *BMI*_1_ and *BMI*_2_ are Body Mass Indexes at the corresponding time points) [21].

A 5% reduction in limb or oedema volumes was recognised as clinically meaningful for BCRL [22]. Sample size calculations were performed based on a previous study evaluating two groups of patients using two specific compression methods (adjustable compression wraps and multi-layer bandaging) [18]. A total of 18 individuals in each intervention group in that study were required to achieve a power of 80%. The treatment comparisons within severity strata in this study are exploratory and underpowered for definitive comparative conclusions.

Patients assessed different compression garments according to the International Compression Club Compression Questionnaire (ICC-CQ)–Patient version [23]. This is the first reliable and validated tool for evaluating different types of compression materials or devices in the upper or lower parts of the body. It consists of:

(1) LE-related symptoms: pain, loss of muscle strength, heaviness, swelling, tight skin, tingling, and fluid leakage (measured on an 11-point Number Rating Scale—NRS);
(2) Garment application and removal difficulties: easy donning, easy doffing (11-point Likert-type NRS scale—from ‘not able at all’ to ‘completely able’);
(3) Comfort in compression: feeling immediately after donning, feeling during daytime, feeling at night, the ability to put clothes over the compression garments, and the appearance of the garment (measured on an 11-point Likert-type scale);
(4) Complications of compression: skin irritation, tender spots, skin damage, itching, warmth, throbbing, cramps, cutting in, slippage, local swelling, bulky feeling, too tight feeling (11-point Likert-type scale—from ‘not at all’ to ‘a lot’);
(5) Physical functioning in relation to compression: able to move wrist, elbow, shoulder, use a spoon, carry out job, complete household chores, practice sports, carry out leisure activities, and social activities (11-point Likert-type scale—from ‘not able at all’ to ‘completely able’).

We used the Polish translation of the ICC-CQ, summing up the numerical results of the responses for the analysis.

In this study, we followed Strengthening the Reporting of Observational Studies in Epidemiology (STROBE) recommendations.

In our study, the primary outcome determines evidence for efficacy of three types of compression used in the treatment. This includes measures such as: affected limb volume, excess volume reduction, joint mobility, and WAC. Secondary outcomes provide supporting evidence of meaningful clinical benefits and safety of the treatment and include: whole physical functioning, lymphedema-related symptoms, compression comfort, and complications. No single endpoint was designated as the sole main efficacy outcome prior to the analysis. 

### Statistical Analysis

The comparison of quantitative variables in three or more groups was performed using the Kruskal–Wallis test, followed by Dunn’s post hoc test if statistically significant differences were detected. For repeated measurements, quantitative variables were compared using the Wilcoxon signed-rank test. The comparison of quantitative variables in three or more repeated measurements was carried out using the Friedman test. When this test revealed significant differences, post hoc analysis was conducted using pairwise Wilcoxon signed-rank tests with a Bonferroni correction. Appropriate effect size measures were reported: *r* for the Wilcoxon signed-rank test, *η*^2^ for the Kruskal–Wallis test and W for Friedman test. A *p*-value of < 0.05 was chosen as the level of statistical significance. All analyses were performed using R (version 4.4.3): A Language and Environment for Statistical Computing (R Foundation for Statistical Computing, Vienna, Austria) [24]. ClinicalTrials.gov Identifier: NCT 07109674. 

## 3. Results

Baseline characteristics—BMI, type of surgery, time since surgery, additional oncotherapeutic modalities, limb and excess volumes—were comparable among women across the three subgroups within the moderate group (MG). Women from the three subgroups within the severe group (SG) were also comparable, except for age and BMI. Specifically, women in the decongestive ACW and MLB subgroups were older than those from the essential ACW subgroup (*p* = 0.011). Additionally, the BMI in the essential subgroup was higher than that in the decongestive ACW subgroup (*p* = 0.005) (Table 1a,b).

### 3.1. Moderate Group (MG)

In the moderate group, a parallel, clinically meaningful reduction in affected limb volume was found after one week of therapy with both types of compression (0.37 L in essential ACWs vs. 0.16 in MLBs, *p* < 0.001). A statistically significant limb volume reduction was also observed in the decongestive ACW group (0.08 L, *p* < 0.002) after one week. The pressure under decongestive ACWs (27 mmHg) was significantly lower compared to essential ACWs (35 mmHg) and MLBs (38 mmHg) (*p* < 0.001).

Affected limb joint mobility (specifically, the ability to move mostly the wrist and elbow, and functional tasks such as using a spoon) was reduced in all patients after two weeks of therapy. Although no significant differences were observed in patient-reported upper-limb mobility between the groups, the patients within the decongestive ACW group rated the affected limb joint mobility higher (not significantly) than those in the essential ACW and MLB groups after the second week. Overall physical functioning in relation to compression decreased in all patients at the end of the first week. LE-related symptoms decreased in all groups; however, this improvement was more pronounced in the MLB group. No significant differences were observed for the perceived comfort associated with wearing compression garments between the groups. All types of compression devices were associated with a certain risk of compression complications. These minor complications occurred less frequently with MLBs than with either type of ACW. These results are found in (Table 2). In the essential ACW subgroup, patients reported: skin irritation (7/9 women), cutting in (6/9 women), local swelling mostly in the hand region (7/9 women), slippage (6/9 women), tightness (5/9 women), and itching (6/9). Women from the decongestive ACW subgroup reported: skin irritation (5/10 women), cutting in (4/10 women), local swelling mostly in the hand region (2/10 women), slippage (4/10 women), tightness (2/10 women), and itching (5/10). In the MLB subgroup, the women reported: skin irritation (5/9 women), cutting in (2/9 women), local swelling mostly in the hand region (1/9 women), slippage (4/9 women), tightness (2/9 women), and itching (5/9).

### 3.2. Severe Group (SG)

In the severe group, a parallel, clinically significant reduction in affected limb volume was found after one week of CDT (0.22 L in decongestive ACWs vs. 0.40 L in essential ACWs vs. 0.36 in MLBs, *p* < 0.001). The pressure under decongestive ACWs (23 mmHg) was significantly lower compared to essential ACWs (35 mmHg) and MLBs (38 mmHg) (*p* < 0.001). Overall physical functioning decreased in all patients; however, in the decongestive ACW group, this decrease occurred only within the first week of therapy. Patients in the decongestive ACW group rated the affected limb joint mobility significantly higher after the second week (*p* = 0.023). LE-related symptoms decreased in all groups; however, this improvement was more pronounced in the MLB group. No significant differences were observed between the groups regarding the perceived comfort of wearing compression devices. All compression types were associated with a certain risk of compression complications, which occurred less frequently with MLB than any type of ACW (Table 3).

Patients in the essential ACW subgroups reported: skin irritation (6/9 women), cutting in (6/9 women), local swelling mostly in the hand region (7/9 women), slippage (6/9 women), tightness (5/9 women), and itching (6/9). Women in the decongestive ACW subgroup reported: skin irritation (5/9 women), cutting in (4/9 women), local swelling mostly in the hand region (1/9 women), slippage (4/9 women), tightness (2/9 women), and itching (5/9). In the MLB subgroup, patients reported: skin irritation (5/9 women), cutting in (2/9 women), local swelling mostly in the hand region (1/9 women), slippage (3/9 women), tightness (2/9 women), and itching (4/9).

## 4. Discussion

Adjustable compression wrap devices with higher stiffness offer an alternative therapy solution for patients who are not able to apply or tolerate multi-layer bandaging. Although ACWs can be considered during the intensive phase of CDT, there is a lack of comprehensive comparative evidence to guide clinicians in selecting between ACWs for moderate and severe stages of BCRL.

To the best of our knowledge, this study is the first preliminary trial to assess the effectiveness of two adjustable compression devices and conventional inelastic bandages in elderly women with moderate (≥20 to <40% PEV) and severe lymphedema (≥40% PEV), focusing on limb volume reduction, physical functioning, and patient comfort.

The present study confirms the efficacy of inelastic ACWs (Circaid^®^ reduction kit and Circaid^®^ juxtafit™ essentials) and MLB in the intensive phase of CDT in moderate and severe lymphedema. In both groups, a parallel clinically significant reduction in affected limb volume and disease-related symptoms was noted after one week of the intensive therapy phase.

There are still few studies describing the effectiveness of ACWs in BCRL survivors [18,19,20]; however, different types of ACW devices with MLB have been compared in the mild [19] to moderate [18] stages of LE (PEV < 20%).

In a randomised trial by Pujol-Blaya et al., the effectiveness of decongestive ACWs was confirmed to be consistent with our findings [18]. Although no significant differences in the reduction in the affected limb were observed between ACWs and MLBs, the treatment in that study was performed exclusively by professional therapists and included manual lymphatic drainage as well [18]. In another study, da Silva et al. indicated that adjustable compression devices (specifically, ‘ready-made’ essential ACWs) and compression sleeves are safe and effective for BCRL patients; however, the authors evaluated only the maintenance phase of physiotherapy [19].

During the initial two weeks of intensive CDT in our study—absolute limb volume reduction served as a useful and more clinically relevant metric for tracking localised changes than excess volume reduction or weight-adjusted changes, and exploratory findings demonstrated a positive trend associated with functional improvement.

Excess volume reduction and weight-adjusted changes are typically reserved for long-term maintenance treatment because they include whole-body size changes and weight fluctuations [21].

The effectiveness of ACWs depends on the dosage (the interface pressure measured in mmHg) as well as the physical and dynamic properties of the textile, such as elasticity and stiffness [25,26,27,28]. Although the optimal pressure under MLBs for the upper limb has been established, the ideal pressure required for ACWs remains unknown.

In the present study, the pressure exerted by the decongestive ACW (Circaid^®^ reduction kit) was significantly lower in both the moderate and severe groups (27 mmHg in the MG vs. 23 mmHg in the SG) in comparison to the essential ACW device (35 mmHg) and MLB (38 mmHg).

It seems that the lower interface pressure under the decongestive ACW device reflected a complex interplay of patient-specific anatomy—including limb shape and tissue characteristics, device properties, and variations in clinical application techniques. According to Laplace’s law, pressure is inversely proportional to the radius of the limb. In more advanced lymphedema such as that observed in our study, irregular limb shapes, localised fibrotic tissue, or prominent bony anatomies can distort pressure readings. Decongestive ACW devices are constructed with panels that conform differently to severe skin folds compared to a continuous, rolled multi-layer bandage, which can bridge across anatomical gaps and create artificial high-pressure points. Decongestive ACW systems often prioritise lower, highly controlled mechanical tension to prevent skin breakdown, whereas MLBs rely on overlapping, multi-component layers that naturally compound pressure according to Laplace’s law. Physiotherapists tend to apply multi-layer bandages tightly to compensate for the rapid, inevitable pressure drop that occurs as oedema reduces. Decongestive wraps are typically applied with more conservative initial tension. Despite these differences in resting pressure, clinical studies show that ACW devices achieve non-inferior or superior volume reduction in lymphedema. Our observations confirm this and indicate that a pressure within the range of 23–27 mmHg in the decongestive ACW was sufficient to obtain positive clinically relevant results.

Despite the higher pressure in the essential ACW and MLB, comfort and tolerance were comparable in all compression system devices and ensured good patient compliance in both the moderate and severe groups.

No interface pressure measurements were conducted under compression in the studies by Pujol-Blaya et al. or da Silva et al. [18,19]. Interface compression pressure is the most critical parameter, and it is essential for the successful treatment of lymphedema. The dosage of compression therapy should be measured at least at the time of application [28,29]. Without pressure measurements, it is impossible to determine whether the compression therapy has been correctly applied [28]. Achieving appropriate pressure appears to be easier with ACWs than with MLBs. The ACW device provides a controlled system for applying and verifying the applied compression force, which guarantees effective self-application and full patient autonomy. Conversely, MLBs are challenging to apply, and elderly BCRL patients may require training and education in this area. Some papers report that many health care professionals fail to apply MLBs with proper pressure [27], especially in more advanced cases of LE. The pressure can be too low and ineffective or too strong, resulting in painful and potentially dangerous compression.

In addition to the pressure, the stiffness of the compression material is crucial for predicting both its effectiveness and patient tolerability [25]. Numerous researchers have demonstrated that higher material stiffness leads to greater improvements in haemodynamic parameters and a more effective reduction in oedema [25,30]. Notably, all compression devices evaluated in our study were inelastic and exhibited a high stiffness index.

Another important aspect of LE therapy is physical functioning while wearing compression devices. Our findings regarding this issue, obtained using the ICC-CQ across three compression modalities, demonstrated that affected limb joint mobility was significantly higher in the decongestive ACWs than in the essential ones or MLBs at the end of the second week in the severe subgroup. Consequently, decongestive ACWs proved to be more functional in daily life compared to other types of compression in severe lymphedema. According to the literature, upper extremity function was lower in patients with LE than in healthy individuals [31]. Reduced mobility and strength can exacerbate swelling and correlate with increased limb volume [7]. Aydin et al. reported that upper limb functional status verified through the DASH (Disabilities of Arm, Shoulder and Hand questionnaire) among BCRL patients was worse in comparison to the patients without LE [31]. In the study by Gultekin et al., significant correlations between lymphedema severity, disease duration and reduced mobility of the upper limb joint were observed [32]. Physiotherapeutic methods involving compression should aim not only to reduce the extent of LE but also to improve arm functionality, thereby enhancing QoL. Better physical functioning in decongestive ACWs in severe lymphedema could be achieved through single-layer ACWs, which are noticeably lighter than MLB. Moreover, the decongestive ACW has the additional option of trimming the material after swelling reduction which minimises fabric overlapping around the elbow and wrist, preventing joint restriction. The absence of this feature in essential ACWs may explain the higher rate of complications—such as slippage and local hand swelling—observed in our previous study [20]. Additionally, the decongestive ACW device maintains sustainable pressure, preventing slippage of the compression which further affects joint mobility. Better joint mobility may partially reflect baseline BMI and limb-volume differences rather than an intrinsic advantage of the decongestive ACW. While the decongestive ACW subgroup demonstrated different functional outcomes, these findings are strictly hypothesis-generating. Due to non-randomised allocation, small sample sizes, and significant baseline disparities in age and BMI, further randomised controlled trials are required to determine whether the decongestive ACW offers an intrinsic functional advantage.

Contradictory results were obtained by da Silva et al., who found comparable functionality using a specific ACW. Our findings suggest that physiotherapy should aim not only to reduce the size of LE, but also to improve or preserve arm functionality, thereby enhancing quality of life among BCRL survivors.

In our study, no further reduction was noted during the self-management phase in the second week. This could be related to the more advanced stage of BCRL and higher BMI observed among the majority of participants. Moderate-to-severe stages of BCRL involve structural skin changes. The continuous expansion of fibrotic tissue and fat deposition alters the thickness of the skin and subcutaneous tissue, leading to tissue hardening [1]. Compression therapy, although highly effective in improving the LE consistency, cannot completely reverse fibrosis in chronic LE. In many studies, higher BMI and obesity are well-documented risk factors for developing and aggravating BCRL due to fat hypertrophy in lymphoedematous tissue and fibrosis [33,34]. BMI in the diagnosis of BCRL is a risk factor associated with severe LE. However, there is no significant association between lymphedema severity and treatments received for breast cancer [33]. High body mass index is correlated with a greater initial lymphedema volume and a lower functional status. Nonetheless, it does not influence the efficacy of CDT in reducing limb volume and improving function [34]. In our study, initial affected limb volumes were similar across subgroups, despite patients in the severe subgroups differing in BMI. Our observations thus suggest that obesity does not undermine the therapeutic effects of CDT. Consequently, CDT based on compression therapy is recommended regardless of the patient’s body mass [34].

Although no additional reduction in limb volume was noted during the second week of the study, compression therapy should be continued to mitigate the risk of inflammatory complications. Patients with more clinically advanced stages of LE are at a particularly high risk of developing cellulitis due to localised immune dysfunction, specifically impaired antigen presentation during lymph transport [35]. The risk of cellulitis increases both with the duration and, importantly, the severity of lymphedema, characterised by the accumulation of adipose tissue and fibrosis [36]. Consequently, best practice guidelines strongly support the usage of compression throughout the course of the disease [2].

Compression therapy is cost-saving in the prevention of recurrent cellulitis among patients with chronic oedema [37]. However, high-quality evidence is still required to confirm its efficacy in LE among breast cancer survivors. We did not evaluate the history of cellulitis in our patient cohort, which may be a limitation of this study; however, the utilisation of diverse compression devices offers patients alternative options beyond standard multi-layer bandaging, especially those with severe LE. These alternatives may increase the positive impact of treatment and success rate resulting from compliance and long-term management.

Despite the fact that women in both the moderate and severe subgroups reported some complications (cutting in, ACW reduction, local hand swelling, skin irritation and slippage in ACW essential) more than in the MLB, no significant differences in comfort between three compression devices were noted. Similar findings have been reported in previous studies [18,19], but in cohorts with less severe stages of the disease (PEV ≥ 10%) [18]. This supports the assumption that therapeutic management in more advanced and chronic cases of LE is inherently more challenging.

A lack of proper education in compression therapy application or prescription can drive non-adherence as patients fail to see its benefits or find it painful and harmful. Selecting the optimal compression therapy for BCRL requires a balance between clinical efficacy and the patient’s daily functional realities. The aspects of usability and patient independence were not directly tested in our study, nor was it assessed which patients may benefit most from each compression type. However, from a practical perspective, our observations indicate that both types of ACW seem to be better options for patients living alone or those lacking access to professional care. This also applies to individuals who are very active in their daily lives and prioritise greater functionality because self-application of ACW seems to be more feasible. Therefore, these patients may feel more independent in daily life and demonstrate improved self-management. MLB is a superior option for patients who have an active, capable caregiver at home or those enrolled in an intensive, clinic-based complex decongestive therapy protocol. Self-application of properly applied MLBs to the upper limb is considered challenging, especially in more advanced lymphedema. These clinical implications should be considered hypotheses for future research related to self-compression therapy.

Several limitations of this study should be acknowledged. First, the sample size of patients who met the inclusion criteria was relatively small. Consequently, the subgroup comparisons are exploratory in nature. Additionally, the study lacked blinding and long-term follow-up. Furthermore, interface pressure was measured exclusively at the beginning of treatment, and the patients’ history of cellulitis was not assessed. Finally, while the Polish version of the ICC-CQ has been formally translated, it has not yet been validated.

A strength of this study is the inclusion of homogeneous patient groups with moderate (PEV ≥ 20 to <40%) and severe (PEV ≥ 40%) BCRL. Moreover, we demonstrated a high level of patient adherence to compression usage within two weeks of applying various therapeutic systems.

## 5. Conclusions

Both adjustable compression wraps may represent feasible alternatives to multi-layer bandaging during the intensive phase of complex decongestive therapy in selected patients. The decongestive adjustable compression wrap demonstrated promising functional advantages in severe lymphedema. The choice between adjustable compression devices and multi-layer bandaging should be guided by patients’ individual needs or preferences. The successful integration of adjustable compression wraps into clinical practice relies on thorough patient training in application techniques to ensure both therapeutic efficacy and safety. Rigorously designed, high-quality, and adequately powered randomised trials with larger sample sizes are required before firm clinical recommendations for breast cancer-related lymphedema can be made.

## Figures and Tables

**Table 1:** (**a**) Patient characteristics in three subgroups of moderate group (MG) before treatment. (**b**) Patient characteristics in three subgroups of severe group (SG) before treatment.

(**a**)					
**Parameter**		**Decongestive ACW-MG** ***N* = 10 **	**Essential ACW-MG** ***N* = 9 **	**MLB-MG** ***N* = 9 **	***p***
Age [years]	Mean (SD)	71.2 (8.2)	65 (9.03)	69.88 (8.24)	*p* = 0.379
	Median (quartile)	69 (66.25–76)	64 (62–72)	71 (65.75–74.75)	
	Range	62–88	48–76	56–81	
	*n*	10	9	8	
BMI [kg/m^2^]	Mean (SD)	33.48 (7.83)	32.77 (6.44)	27.54 (7.26)	*p* = 0.199
	Median (quartile)	30.75 (28.27–39.68)	31.6 (29.1–36.7)	24 (23.4–31.2)	
	Range	21.3–45.6	23.8–43.2	20–43.3	
	*n*	10	9	9	
Dominant limb affected	Yes	7 (70.00%)	4 (44.44%)	5 (55.56%)	*p* = 0.569
	No	3 (30.00%)	5 (55.56%)	4 (44.44%)	
Months since surgery	Mean (SD)	115.6 (81.66)	69.11 (83.62)	66.67 (51.56)	*p* = 0.281
	Median (quartile)	119.5 (37.25–191.5)	38 (18–87)	31 (29–121)	
	Range	12–224	6–267	5–129	
	*n*	10	9	9	
Type of surgery	BCT	4 (40.00%)	4 (44.44%)	1 (11.11%)	*p* = 0.351
	M	6 (60.00%)	5 (55.56%)	8 (88.89%)	
Axillary node dissection	No	0 (0.00%)	0 (0.00%)	0 (0.00%)	*p* = 1
	Yes	10 (100.00%)	9 (100.00%)	9 (100.00%)	
Chemotherapy	No	2 (20.00%)	0 (0.00%)	4 (44.44%)	*p* = 0.08
	Yes	8 (80.00%)	9 (100.00%)	5 (55.56%)	
Axillary irradiation	No	1 (10.00%)	1 (11.11%)	3 (33.33%)	*p* = 0.473
	Yes	9 (90.00%)	8 (88.89%)	6 (66.67%)	
Breast irradiation	No	1 (10.00%)	1 (11.11%)	3 (33.33%)	*p* = 0.473
	Yes	9 (90.00%)	8 (88.89%)	6 (66.67%)	
Hormonotherapy	No	3 (30.00%)	2 (22.22%)	3 (33.33%)	*p* = 1
	Yes	7 (70.00%)	7 (77.78%)	6 (66.67%)	
Immunotherapy	No	10 (100.00%)	8 (88.89%)	9 (100.00%)	*p* = 0.643
	Yes	0 (0.00%)	1 (11.11%)	0 (0.00%)	
Affected limb volume [mL]	Mean (SD)	3235.18 (1036.69)	3556.84 (875.28)	3260.51 (1061.42)	*p* = 0.541
	Median (quartile)	3075.7 (2560.15–3597.6)	3483.8 (3024.3–3583)	2893.7 (2747.9–3433.2)	
	Range	2040.7–5437.1	2582.2–4972.7	2340.7–5912.9	
	*n*	10	9	9	
Opposite limb volume [mL]	Mean (SD)	2538.7 (780.58)	2874.18 (728.06)	2613.78 (960.04)	*p* = 0.504
	Median (quartile)	2427.45 (2066.38–2847.1)	2858 (2430.8–3247.3)	2232.3 (2196.6–2788.1)	
	Range	1542–4100.1	2003.4–4081.3	1765.7–4998	
	*n*	10	9	9	
Excess volume [%]	Mean (SD)	27.24 (3.23)	24.29 (7.42)	26.01 (4.81)	*p* = 0.629
	Median (quartile)	26.8 (25.25–28.3)	25.37 (20.71–28.89)	24.97 (23.14–30.74)	
	Range	22.5–32.6	9.68–33.24	18.31–32.56	
	*n*	10	9	9	
(**b**)					
**Parameter**		**Decongestive ACW-SG** ***N* = 9 **	**Essential ACW-SG** ***N* = 9 **	**MLB-SG** ***N* = 9 **	***p***
Age [years]	Mean (SD)	76.89 (13.82)	59.67 (9.04)	70.89 (11.76)	*p* = 0.011 dec. MLB > ess
	Median (quartile)	72 (69–88)	60 (57–67)	72 (63–77)	
	Range	53–96	45–69	50–90	
	*n*	9	9	9	
BMI [kg/m^2^]	Mean (SD)	25.76 (3.89)	33.04 (5.19)	28.97 (2.96)	*p* = 0.005 ess. > dec
	Median (quartile)	25.6 (22.2–28.3)	30.4 (29.6–34.8)	28.3 (28.1–29.6)	
	Range	20.3–32	28.8–44.8	24–34.2	
	*n*	9	9	9	
Dominant limb affected	Yes	5 (55.56%)	6 (66.67%)	5 (55.56%)	*p* = 1
	No	4 (44.44%)	3 (33.33%)	4 (44.44%)	
Months since surgery	Mean (SD)	217.22 (162.29)	110.57 (125.02)	120.62 (171.85)	*p* = 0.239
	Median (quartile)	189 (103–318)	63 (39.5–131)	19 (11.5–190.75)	
	Range	7–516	9–361	6–480	
	*n*	9	7	8	
Type of surgery	BCT	0 (0.00%)	3 (33.33%)	2 (22.22%)	*p* = 0.314
	M	9 (100.00%)	6 (66.67%)	7 (77.78%)	
Axillary node dissection	No	0 (0.00%)	0 (0.00%)	0 (0.00%)	*p* = 1
	Yes	9 (100.00%)	9 (100.00%)	9 (100.00%)	
Chemotherapy	No	3 (33.33%)	0 (0.00%)	1 (11.11%)	*p* = 0.28
	Yes	6 (66.67%)	9 (100.00%)	8 (88.89%)	
Axillary irradiation	No	2 (22.22%)	1 (11.11%)	1 (11.11%)	*p* = 1
	Yes	7 (77.78%)	8 (88.89%)	8 (88.89%)	
Breast irradiation	No	2 (22.22%)	1 (11.11%)	1 (11.11%)	*p* = 1
	Yes	7 (77.78%)	8 (88.89%)	8 (88.89%)	
Hormonotherapy	No	3 (33.33%)	1 (11.11%)	3 (33.33%)	*p* = 0.632
	Yes	6 (66.67%)	8 (88.89%)	6 (66.67%)	
Immunotherapy	No	9 (100.00%)	6 (66.67%)	8 (88.89%)	*p* = 0.28
	Yes	0 (0.00%)	3 (33.33%)	1 (11.11%)	
Affected limb volume [ml]	Mean (SD)	3010.71 (664.83)	3885.69 (907.56)	3317.26 (521.31)	*p* = 0.11
	Median (quartile)	2942.5 (2403–3564)	3827.7 (3257.5–4325)	3403.2 (2985.9–3508.1)	
	Range	2079.9–3877.2	2740.5–5664.4	2563–4248.9	
	*n*	9	9	9	
Opposite limb volume [ml]	Mean (SD)	1978.96 (479.16)	2584.96 (536.75)	2326.08 (429.97)	*p* = 0.05
	Median (quartile)	2019 (1586.4–2257.8)	2528.7 (2146–2779)	2444 (2137.4–2501.4)	
	Range	1233.1–2867.7	1917.6–3544.5	1676.1–3171.4	
	*n*	9	9	9	
Excess volume [%]	Mean (SD)	53.3 (13.3)	49.76 (8.05)	43.44 (8.64)	*p* = 0.188
	Median (quartile)	51.5 (45.7–65)	51.37 (42.91–55.63)	40.25 (38.7–52.91)	
	Range	35.2–71.4	35.98–59.81	33.98–56.91	
	*n*	9	9	9	

*p*—Categorical variables: chi-square test or Fisher’s exact test. (**a**) Quantitative variables: Kruskal–Wallis test; (**b**) Quantitative variables: Kruskal–Wallis test + post hoc analysis (Dunn’s test).

**Table 2:** Results in moderate group (MG).

Parameter	Decongestive ACW-MG (Median, IQR) *N* = 10	Essential ACW-MG (Median, IQR) *N* = 9	MLB-MG (Median, IQR) *N* = 9	Subgroup Difference	*p*
Interface pressure under compression (mmHg)	27 (21–29)	35 (30–40)	38 (35–40)		*p* < 0.001 η^2^ = 0.664
Affected limb volume (L)					
T0 before therapy	3.07 (2.56–3.60)	3.48 (3.02–3.58)	2.89 (2.75–3.43)		*p* = 0.541 η^2^ = −0.031
T1 after 1 week	2.99 (2.44–3.36)	3.11 (2.70–3.44)	2.73 (2.47–2.86)		*p* = 0.662 η^2^ = −0.047
T2 after 2 weeks	2.99 (2.55–3.15)	3.07 (2.8–3.50)	2.69 (2.47–2.86)		*p* = 0.419 η^2^ = −0.01
	T0 > T1,T2	T0 > T1,T2	T0 > T1,T2		
	*p* = 0.002 W = 0.63	*p* = 0.001 W = 0.753	*p* = 0.001 W = 0.778		
Excess volume reduction (%)					
after 1 week	−6.75 (−8.60–−4.18)	−6.29 (−8.42–−0.72)	−11.5 (−14.56–−11.14)	dec, ess > MLB	*p* = 0.003 η^2^ = 0.376
after 2 weeks	−3.50 (−9.60–−2.48)	−8.90 (−10.42–−3.94)	−15.67 (−16.97–−10.49)	dec, ess > MLB	*p* = 0.032 η^2^ = 0.196
	*p* = 0.492 r = 0.419	*p* = 0.605 r = 0.968	*p* = 0.666 r = 0.928		
WAC after 2 weeks (%)	−3.76 (−8.34–−1.96)	−6.75 (−8.63–−4.40)	−8.61 (−11.63–−6.84)		*p* = 0.12 η^2^ = 0.09
Patient-reported upper-limb mobility (NRS)					
T0 before therapy	25.5 (18.25–27.75)	25.0 (22.0–30.0)	29.0 (26.0–30.0)		*p* = 0.389 η^2^ = −0.004
T1 after 1 week	20.0 (16.50–24.00)	12.0 (10.0–18.0)	17.0 (16.0–21.0)		*p* = 0.389 η^2^ = −0.004
T2 after 2 weeks	18.0 (11.50–20.75)	10.0 (6.0–14.0)	11.0 (5.0–24.0)		*p* = 0.306 η^2^ = 0.015
	T0 > T2	T0 > T2	T0 > T2		
	*p* = 0.008 W = 0.478	*p* = 0.002 W = 0.61	*p* = 0.036 W = 0.369		
Total physical functioning in relation to compression (NRS)					
T0 before therapy	63.0 (52.25–69.75)	57.0 (51.0–69.0)	60.0 (50.0–64.0)		*p* = 0.918 η^2^ = −0.073
T1 after 1 week	29.0 (20.5–30.75)	24.0 (19.0–30.0)	40.0 (18.0–45.0)		*p* = 0.344 η^2^ = 0.005
T2 after 2 weeks	22.5 (17.75–30.75)	25.0 (15.0–34.0)	29.0 (20.0–39.0)		*p* = 0.673 η^2^ = −0.048
	T0 > T1,T2	T0 > T2	T0 > T2		
	*p* = 0.002 W = 0.61	*p* = 0.003 W = 0.642	*p* = 0.008 W = 0.531		
LE-related symptoms (NRS)					
T0 before therapy	25.0 (22.5–33.0)	18.0 (8.0–23.0)	25.0 (18.0–28.0)		*p* = 0.104 η^2^ = 0.101
T1 after 1 week	21.5 (12.25–24.75)	15.0 (12.0–22.0)	11.0 (3.0–14.0)		*p* = 0.11 η^2^ = 0.096
T2 after 2 weeks	23.5 (17.5–27.5)	13.0 (7.0–22.0)	10.0 (2.0–12.0)	dec > MLB	*p* = 0.007 η^2^ = 0.316
	T0 > T1		T0 > T1,T2		
	*p* = 0.014 W = 0.43	*p* = 0.124 W = 0.232	*p* = 0.002 W = 0.704		
Comfort of compression (NRS)					
after 1 week	29.5 (24.75–32.25)	25.0 (22.0–28.0)	25.0 (21.0–35.0)		*p* = 0.438 η^2^ = −0.014
after 2 weeks	28.5 (21.5–33.0)	38.0 (27.0–42.0)	31.0 (28.0–34.0)		*p* = 0.429 η^2^ = −0.012
	*p* = 0.941 r = 0.032	*p* = 0.074 r = 0.612	*p* = 0.516 r = 0.237		
Complications of compression (NRS)					
after 1 week	25.5 (19.5–30.75)	20.0 (18.0–36.0)	8.0 (6.0–23.0)	dec. ess. > MLB	*p* = 0.032 η^2^ = 0.195
after 2 weeks	30.5 (18.25–38.5)	35.0 (22.0–46.0)	11.0 (3.0–17.0)	dec. ess. > MLB	*p* = 0.015 η^2^ = 0.255
	*p* = 0.73 r = 0.145	*p* = 0.18 r = 0.474	*p* = 0.789 r = 0.079		

Effect size measures for the tests used: W—Friedman test (Wilcoxon with Bonferroni post hoc correction); η^2^—Kruskal–Wallis test; r—Wilcoxon signed-rank test.

**Table 3:** Results in severe group (SG).

Parameter	Decongestive ACW-SG (Median, IQR) *N* = 9	Essential ACW-SG (Median, IQR) *N* = 9	MLB SG (Median, IQR) *N* = 9	Subgroup Difference	*p*
Interface pressure under compression (mmHg)	23 (21.2–24)	35 (30–40)	38 (36–40)		*p* < 0.001 η^2^ = 0.621
Affected limb volume (L)					
T0 before therapy	2.94 (2.40–3.56)	3.83 (3.26–4.32)	3.40 (2.98–3.50)		*p* = 0.11 η^2^ = 0.101
T1 after 1 week	2.72 (2.31–3.28)	3.43 (2.98–4.05)	3.04 (2.62–3.19)		*p* = 0.104 η^2^ = 0.105
T2 after 2 weeks	2.70 (2.30–3.25)	3.42 (2.91–3.95)	2.88 (2.60–3.13)		*p* = 0.051 η^2^ = 0.165
	T0 > T1, T2	T0 > T1, T2	T0 > T1, T2		
	*p* = 0.001 W = 0.827	*p* = 0.005 W = 0.593	*p* = 0.001 W = 0.827		
Excess volume reduction (%)					
after 1 week	−8.7 (−18.3–−5.3)	−12.54 (−20.33–−7.91)	−13.11 (−14.61–−7.48)		*p* = 0.797 η^2^ = −0.064
after 2 weeks	−10.4 (−14.5–−7.5)	−15.8 (−17.18–−10.38)	−14.73 (−18.22–−11.37)		*p* = 0.62 η^2^ = −0.044
	*p* = 0.666 r = 0.928	*p* = 0.863 r = 0.810	*p* = 0.666 r = 0.928		
WAC after 2 weeks (%)	−6.94 (−8.82–−3.77)	−7.77 (−8.10–−6.77)	−10.64 (−12.76–−6.20)		*p* = 0.459 η^2^ = −0.019
Patient-reported upper-limb mobility (NRS)					
T0 before therapy	25.0 (22.0–28.0)	22.0 (21.0–27.0)	23.0 (22.0–25.0)		*p* = 0.766 η^2^ = −0.061
T1 after 1 week	24.0 (17.0–25.0)	15.0 (11.0–22.0)	21.0 (12.0–22.0)		*p* = 0.274 η^2^ = 0.024
T2 after 2 weeks	17.0 (9.0–25.0)	8.0 (7.0–10.0)	7.0 (5.0–8.0)	dec. > ess. MLB	*p* = 0.023 η^2^ = 0.231
	T0 > T2	T0, T1 > T2	T0 > T2		
	*p* = 0.048 W = 0.337	*p* = 0.001 W = 0.784	*p* = 0.018 W = 0.444		
Total physical functioning in relation to compression (NRS)					
T0 before therapy	58.0 (44.0–64.0)	51.0 (47.0–58.0)	51.0 (49.0–52.0)		*p* = 0.677 η^2^ = −0.051
T1 after 1 week	36.0 (26.0–40.0)	36.0 (24.0–48.0)	38.0 (36.0–47.0)		*p* = 0.611 η^2^ = −0.042
T2 after 2 weeks	27.0 (18.0–41.0)	23.0 (19.0–32.0)	21.0 (8.0–26.0)		*p* = 0.43 η^2^ = −0.013
	T0 > T1, T2	T0, T1 > T2	T0 > T2		
	*p* = 0.002 W = 0.695	*p* = 0.001 W = 0.827	*p* = 0.042 W = 0.352		
LE-related symptoms (NRS)					
T0 before therapy	32.0 (16.0–34.0)	37.0 (23.0–47.0)	22.0 (17.0–23.0)		*p* = 0.122 η^2^ = 0.092
T1 after 1 week	21.0 (14.0–30.0)	13.0 (12.0–20.0)	9.0 (4.0–12.0)		*p* = 0.070 η^2^ = 0.138
T2 after 2 weeks	15.0 (5.0–30.0)	14.0 (10.0–21.0)	2.0 (0–4.0)	dec. ess. > MLB	*p* = 0.009 η^2^ = 0.312
	T0 > T2	T0 > T2	T0 > T1, T2		
	*p* = 0.010 W = 0.513	*p* = 0.003 W = 0.642	*p* = 0.001 W = 0.827		
Comfort of compression (NRS)					
after 1 week	42.0 (26.0–45.0)	28.0 (26.0–31.0)	30.0 (19.0–40.0)		*p* = 0.177 η^2^ = 0.061
after 2 weeks	37.0 (33.0–42.0)	37.0 (21.0–46.0)	37.0 (22.0–41.0)		*p* = 0.859 η^2^ = −0.071
	*p* = 0.844 r = 0.079	*p* = 0.359 r = 0.336	*p* = 0.91 r = 0.059		
Complications of compression (NRS)					
after 1 week	27.0 (21.0–40.0)	29.0 (20.0–34.0)	7.0 (4.0–11.0)	dec. ess. > MLB	*p* = 0.001 η^2^ = 0.481
after 2 weeks	23.0 (16.0–28.0)	15.0 (13.0–31.0)	4.0 (4.0–11.0)	dec. ess > MLB	*p* = 0.021 η^2^ = 0.239
	*p* = 0.301 r = 0.375	*p* = 0.188 r = 0.454	*p* = 0.719 r = 0.197		

Effect size measures for used tests: W—Friedman test (Wilcoxon with Bonferroni post hoc correction); η^2^—Kruskal–Wallis test; r—Wilcoxon signed-rank test.

## Data Availability

Research Data has been published in Faculty of Rehabilitation and can be found at https://akf.rodbuk.pl/dataset.xhtml?persistentId=doi:10.58145/AKF/I1C5GU; https://doi.org/10.58145/AKF/I1C5GU; https://akf.rodbuk.pl/dataverse/fr (accessed on 12 September 2026).

## Abbreviations

The following abbreviations are used in this manuscript:

BCRL	Breast cancer-related lymphedema
ISL	International Society of Lymphology
LE	Lymphedema
PEV	Percentage of excess volume
CT	Compression therapy
MLB	Multi-layer bandaging
CG	Compression garments
CDT	Complex Decongestive Therapy
QoL	Quality of life
ACW	Adjustable Compression Wraps
WAC	Weight-adjusted change
BMI	Body mass index
ICC-CQ	International Compression Club Compression Questionnaire
